# PLA Feedstock Filled with Spent Coffee Grounds for New Product Applications with Large-Format Material Extrusion Additive Manufacturing

**DOI:** 10.1021/acsomega.3c05669

**Published:** 2024-02-01

**Authors:** Martina Paramatti, Alessia Romani, Gianluca Pugliese, Marinella Levi

**Affiliations:** † Department of Chemistry, Materials, and Chemical Engineering “Giulio Natta”, Piazza Leonardo Da Vinci 32, 20133 Milano, Italy; ‡ Design Department, Via Durando, 20158 Milano, Italy; § LowPoly SL, Avenida Real de Pinto 91, Nave 07, 28021 Madrid, Spain

## Abstract

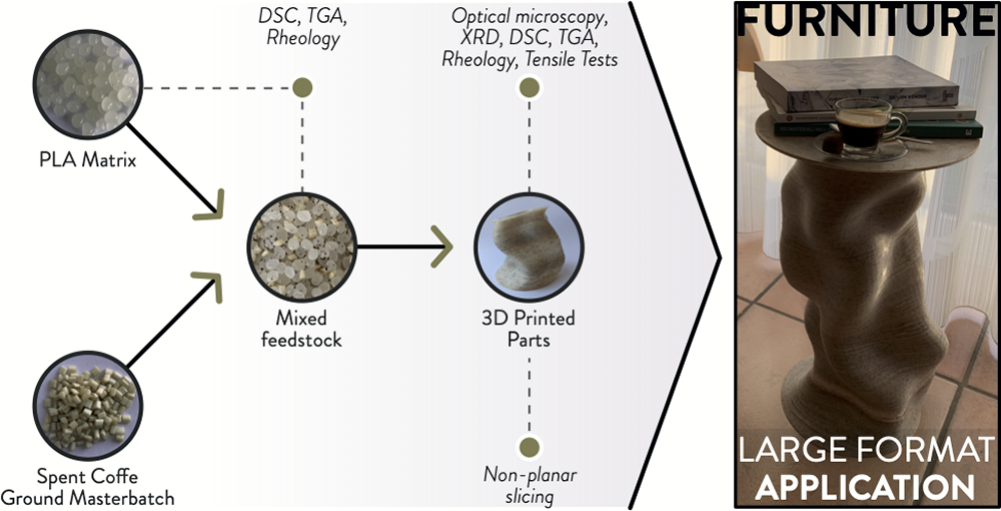

Food waste and loss generate significant waste such as spent coffee grounds (SCGs) from coffee consumption. These byproducts can be valorized by following circular economy and bioeconomy principles, e.g., using SCGs in polymer-based composites for 3D printing. Although desktop-size material extrusion additive manufacturing is increasingly adopted for biomass-polymer-based composites, the potential of large-format direct extrusion 3D printing systems remains unexplored. This work investigated the thermal, rheological, and mechanical properties of PLA/SCG composites for applications with a large-format pellet extrusion 3D printer. The formulations exhibit minimal degradation at typical 3D printing temperatures of PLA, i.e., ∼190 °C, and limited effects on crystallinity by increasing the SCG weight percentage. The decrease in viscosity due to SCGs improves the printability and layer adhesion, as confirmed by the tensile test results, such as higher ultimate tensile strength and elongation at break values compared to those of the state-of-the-art values. Using pellet feedstocks contributes to limiting the effects of thermomechanical degradation by reducing raw material processing, i.e., avoiding filament extrusion. Using PLA/SCGs formulations was demonstrated through 3D printed complex parts with nonplanar slicing techniques, including a large-scale furniture product, validating large-format pellet extrusion 3D printers for scaling up the use of biomass-filled polymers.

## Introduction

In recent years, awareness regarding food waste and loss has been increased with different initiatives to address this global issue, i.e., the United Nations Sustainable Development Goals (SDGs).^1^ Reducing food waste and loss requires examining the entire supply chain from primary production to consumption and disposal. Food waste leads to significant environmental, social, and economic impacts, increasing greenhouse gas emissions, nutrient loss, and hunger.^2^ For instance, the resources involved in food waste and loss are estimated to result in a carbon footprint of 3.3 billion tons of CO_2_.^2^

Coffee consumption represents a well-established market, with a global daily consumption of around 3 billion cups.^3^ Waste generation and environmental impact are significant concerns, particularly regarding spent coffee grounds (SCGs), packaging materials, and single-use coffee pods.^3,4^ As SCGs can potentially become environmental pollutants, SCGs byproducts can be valorized by collecting and recycling them, i.e., as food and drug ingredients, in bioenergy recovery, for compost and fertilizers, and as secondary raw material.^5,6^ Valorizing SCGs aligns with the concepts of circular economy and bioeconomy for value retention of resource flows. Circular economy addresses global challenges, such as climate change, biodiversity loss, and pollution, through waste elimination, product and material circulation, and nature regeneration.^7^ The values of resource and energy flows are retained through time thanks to different strategies, i.e., reuse, repair, and recycling.^8^ Similarly, bioeconomy utilizes renewable biological resources to produce food, materials, and energy, highlighting possible interrelationships with circular economy for sustainable biomass utilization and resource management, i.e., through industrial symbiosis.^9,10^

Focusing on circular material flows, SCGs can be used as filler materials, reducing waste generation through new secondary raw materials, i.e., polymer-based composites. Polyethylene (PE) and polypropylene (PP) filled with SCGs are commonly used in packaging applications due to their cost-effectiveness and properties, such as increased impact strength and stiffness.^11,12^ Polylactic acid (PLA) represents an affordable alternative to fossil-based thermoplastics, improving biodegradability rates when combined with SCGs. PLA/SCGs composites show potential uses in food packaging and as feeding material for 3D printing.^13,14^

Additive manufacturing (AM) fabricates three-dimensional parts by adding material layer by layer. Thanks to their versatility and accessibility, material extrusion AM processes are widely adopted in real-world contexts.^15^ Fused deposition modeling (FDM, or fused filament fabrication, FFF) selectively deposits melted materials from filament feedstocks through a nozzle, and it is usually associated with thermoplastics and thermoplastic-based composites for small-format systems.^16,17^ However, the scale of 3D printers varies, ranging from desktop-sized units using spooled filaments to large-format AM (LFAM) machines employing a single-screw pellet extrusion system. Pellet extrusion systems, i.e., Fused granular fabrication (FGF, or fused granular fabrication, FPF), offer cost advantages and the ability to blend different polymers or use recycled materials and byproducts, reducing the processing steps to create raw materials, i.e., filament extrusion.^18−20^ Despite the benefits of using LFAM and FGF for new applications with secondary raw materials,^21^ most works have studied biomass waste materials on desktop-size 3D printers, including PLA/SCGs filaments. From the literature, LFAM systems are still scarcely considered as potential scaling up of polymer-based composites with biomass byproducts.^22^

This work studies a biobased polymer composite filled with biomass byproducts from food processing, i.e., SCGs, for applications with a LFAM pellet extrusion system. PLA-based formulations with SCGs from 0 to 10 wt % were selected for the characterizations and the 3D printing tests. The thermal behavior of the feedstocks and 3D printed samples was evaluated through thermogravimetric analysis (TGA) and differential scanning calorimetry (DSC) tests. The influence of SCGs on PLA crystallization was studied through X-ray diffraction (XRD). The rheological behavior was investigated through flow stress ramp tests. Tensile tests were performed to study the mechanical behavior of the different formulations. Optical microscopy images were used to qualitatively assess the morphology of the 3D printed samples and the fracture cross sections of the tensile specimens. Some samples were fabricated with conventional and nonplanar slicing techniques to demonstrate the use of PLA/SCGs feedstocks for complex geometries, and a demo product was designed and 3D printed as a proof-of-concept for real-world applications, i.e., furniture. Biomass-based composites, such as PLA/SCGs, represent a valuable option for new applications with LFAM to foster circular bioeconomy strategies based on material flows.

## Materials and Methods

### Materials

The SCG polymer composites were obtained by mixing PLA pellet feedstock and the SCGs in the form of masterbatch. Spent coffee grounds (SCGs) were provided by LowPoly SL (Madrid, Spain). This coffee masterbatch (CM) is composed of a PLA carrier with a dispersant and dehydrated SCGs, facilitating the powder dispersion into the matrix. PLA Ingeo Biopolymer 3D850 from NatureWorks LCC (Minneapolis, MN, USA) was selected as the matrix of the SCGs-based formulations. The materials were used as received and mixed to obtain the feedstock for the large-format FGF 3D printer without requiring extrusion of the 3D printing filaments. Tests were performed on five material formulations: (i) PLA pellet matrix as a benchmark (PLA), (ii) coffee masterbatch pellet (CM), (iii) 3D printed PLA matrix as a benchmark (100PLA0CM), (iv) 3D printed PLA with 5 wt % of CM (95PLA5CM), and (v) 3D printed PLA with 10 wt % of CM (90PLA10CM).

### Experimental Methods

**Scheme 1:**
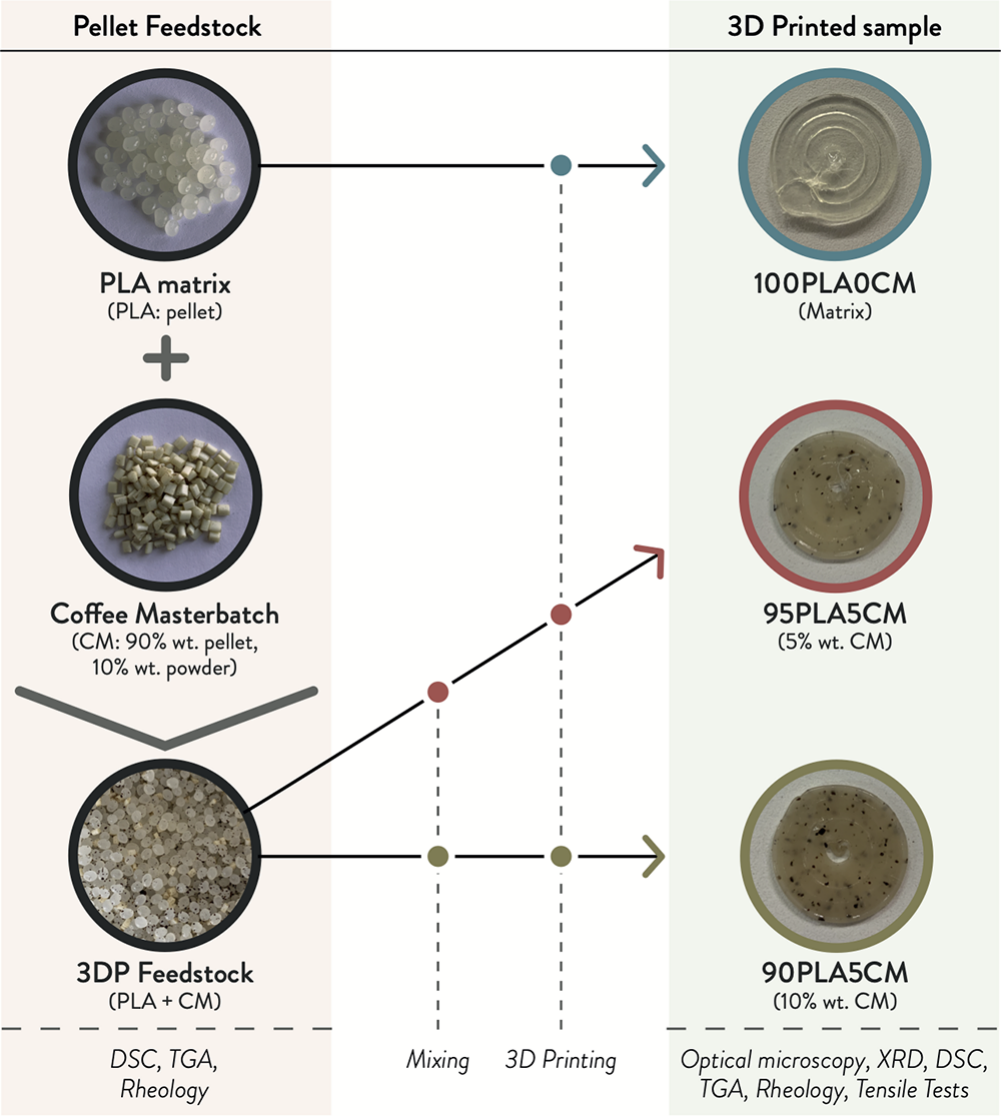
Resume of the Experimentation Conducted in This Work with the Different Material Formulations (PLA Pellet; CM Pellet; 100PLA0CM; 95PLA5CM; 90PLA10CM), Processing Steps, and Characterization Tests

Scheme 1 resumes the workflow of the experimentation, including the different characterizations performed on the PLA/SCGs formulations as pellet feedstocks or 3D printed samples.

The PLA/SCGs formulations were 3D printed with a Delta Wasp 3MT Industrial (Wasp S.r.l., Massa Lombarda, Italy), a large-format fused granular fabrication (FGF) AM system with a closed chamber, and a single screw pellet extruder for direct feeding. The extruder is equipped with a stainless steel nozzle of 3 mm diameter, which represents a typical dimension for this specific 3D printer and, in general, similar LFAM systems. The 3D printer uses an aluminum circular building plate and has a maximum building volume of 1000 mm in diameter and 1200 mm in height. Its 3D printing volume is ∼1 m^3^, the typical value for LFAM apparatuses.^18^

Three different types of samples were fabricated according to the parameters of Table 1: characterization samples, 3D printing samples, and product application. The characterization samples were produced for the tensile tests (ASTM D638-22 Type I shape), XRD analysis (rectangular shape), and rheological measurements (cylindric shape). They were designed with Solidworks (Dassault Systèmes, Vélizy-Villacoublay, France), and the gcode files were prepared with Simplify3D (Simplify3D, Cincinnati, OH, US), using OctoPrint to monitor the 3D printing process.^23^ 3D printing samples were 3D printed to demonstrate the feasibility of complex shapes with the 90PLA10CM formulation. The two sets of samples have a nominal dimension of 90 mm × 90 mm × 90 mm, with maximum overhangs of 30°. Conventional (planar) and nonplanar slicing techniques were used to produce the samples,^24^ reaching a maximum curvature angle of the layers of 32°. Further details on the sample batches can be found in the Supporting Information. A coffee table with nonplanar slicing features was designed and 3D printed as a potential application (maximum nominal size of 300 × 300 × 450 mm). The samples were designed and sliced with Grasshopper on Rhinoceros (Robert McNeel & Associates, Seattle, WA, USA).^25^

**Table 1:** Slicer Parameters of the 3D Printed Samples

parameters	unit	characterization samples (tensile tests, XRD, rheology)	3D printing samples	product application (furniture)
coffee masterbatch %	wt %	0, 5, 10	10	10
curvature angle	°	0	0–32	0–20
lyer height	mm	0.5	0.5, 0.7, 1, 1.2	1
feed rate	mm/s	20	10, 15, 20, 30	10
T extruder	°C	190	190	190
T bed	°C	80	80	75
extrusion multiplier	/	1.8	2	3
extrusion width	/	3.8^a^	/	/
perimeters	/	1	1	1
top layer	/	4	0	0
bottom layer	/	4	1	0
infill percentage and pattern	%	100, rectilinear^b^	0, 20, grid	0, //
external infill angle offsets	°	180	/	/
internal infill angle offsets	°	45, −45	45, −45	/
outline overlap	%	50	10	/
skirt layer	/	1	1	1
skirt offset	mm	20	20	20
skirt outlines	/	3	3	3

a The extrusion width parameter was changed to 3.6 only for the XRD samples.

b The infill pattern was set to circular for the rheology samples.

Thermogravimetric analysis (TGA) tests were performed using the TGA Q500 machine (TA Instruments Inc., New Castle, DE, US) by analyzing in an inert atmosphere (nitrogen) the thermal decomposition of 10–20 mg samples of PLA pellet, CM, and the three 3D printed formulations.^26^ Thermal properties of all samples were assessed by TGA with a temperature range from 25 to 800 °C and a heating rate of 10 °C/min.

Differential scanning calorimetry (DSC) tests were conducted on a Mettler-Toledo DSC/823e machine (Mettler Toledo, Columbus, OH, US) with 10–20 mg samples of PLA pellet, CM, and the three 3D printed formulations. Three thermal cycles (heating–cooling–heating) were carried out at scan rates of 20 °C/min: a first heating scan from 25 to 220 °C, a cooling scan from 220 to −50 °C, and a second heating scan from −50 to 220 °C. These steps were useful to evaluate the glass transition temperature (*T*_g_), the cold crystallization temperature (*T*_cc_), the enthalpy of cold crystallization (Δ*H*_cc_), the melting temperature (*T*_m_), and the enthalpy of fusion (Δ*H*_m_).

X-ray diffraction (XRD) measurements were used to investigate the influence of SCGs on PLA crystallization by comparing the 3D printed samples (18 × 30 × 3 mm). The tests were performed on a Bruker D8 Advance diffractometer (Bruker, Billerica, MA, US), with a wavelength λ = 1.5406 Å, collecting the diffraction data from 0 to 80° of 2Θ, with 0.02°of step size and 4 s of time per step.

The thermal characterization analyses were conducted on batches made of three samples for each material composition to assess the repeatability of the tests. The mean values were then calculated by starting from the results from each test.

Rheological tests were performed to simulate the processing conditions of the extruder chamber. Since the mixing process of PLA and CM directly occurs in the 3D printing extruder chamber, tests were done on PLA pellet, CM, and 3D printed cylindrical samples (⌀22 × 1 mm) of the three formulations. Flow ramp tests were performed on a Discovery HR-2 hybrid rheometer (TA Instruments Inc., New Castle, DE, US) with a 25 mm parallel steel plate geometry. Tests were carried out at 190 °C for 180 s, with an applied shear rate ranging from 10^–2^ to 10^2^ s^–1^. The gap was set at 300 μm for PLA pellets, 700 μm for CM, and 1000 μm for the 3D printed samples. The results were used to calculate the zero shear viscosity (η_0_) and the viscosity values when the shear rate is 27.5 s^–1^, approximating the flow behavior at a reference shear rate corresponding to typical 3D printing conditions, i.e., 20 mm/s.^27^ The shear rate was calculated according to eq 1:
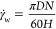
1where γ̇_w_ is the expected shear rate in the internal screw channel wall, *D* the screw diameter in mm, *N* the screw speed in rps, and *H* the channel depth in mm.^28^

Tensile tests were performed with a ZwickRoell Z010 testing machine (ZwickRoell GmbH & Co. KG, Ulm, Germany) equipped with a 10 kN load cell and square grips at 1 mm/min. At least five samples for each composition were tested using the Type I dog bone shape of ASTM D638-22 standard.^29^ Specimens had a nominal gauge length of 57 mm, a thickness of 3 mm, and a width of 13 mm. The experimental stress–strain curves were used to calculate the mean values and standard errors of elastic modulus (*E*), ultimate tensile strength (σ_m_), fracture strength (σ_b_), elongation at maximum strength (ε_m_), and elongation at break (ε_b_).

Optical microscopy was used to qualitatively evaluate the morphology of the sample surface and the fracture cross-sectional surface of tensile specimens, i.e., grain dimensions and porosity. The tests were conducted on an Olympus BX60 optical microscope (Olympus, Shinjuku-ku, Tokyo, Japan) with a magnification objective lens set at 50×.

## Results and Discussion

### Thermal Properties

TGA analysis was performed to study the thermal stability of PLA pellet, CM, and composite formulations (Figure S1, Supporting Information). According to the onset temperature (*T*_onset_) of Table 2, the CM degrades at lower temperatures than the PLA granulated sample (∼38 °C less). This behavior is confirmed by the 3D printed material formulations, which show a decreasing trend as the CM percentage increases (−15.5% between 100PLA0CM and 90PLA10CM), in agreement with similar studies with SCGs,^5^ also on small-scale 3D printers.^13,30^ The 3D printed samples show lower thermal stability as a function of CM loading, which triggers material degradation at lower temperatures. Despite this decreasing trend, the samples show little or no degradation at *T* = 190°. Accordingly, the PLA/SCGs formulations are thermally stable during the 3D printing process at typical extrusion temperatures of PLA and PLA-based biomass composites.

**Table 2:** Thermal Properties of PLA Pellet, CM, and the 3D Printed Formulations

sample		TGA		DSC (2nd heating scan)			
		*T*_onset_ (°C)	*T*_g_ (°C)	*T*_cc_ (°C)	Δ*H*_cc_ (J/g)	*T*_m_ (°C)	Δ*H*_m_ (J/g)
granulated material (pellet)	PLA	296.3	61.8	/	/	/	/
	CM	257.7	61.8	/	/	121.2	6.9
3D printed material	100PLA0CM	302.4	61.2	133.6	0.6	154.2	1.9
	95PLA5CM	262.6	61.8	131.1	8	154.7	10.6
	90PLA10CM	255.5	59.5	130	6.8	154	8.3

Table 2 summarizes the results of the DSC analysis, i.e., glass transition temperature (*T*_g_), cold crystallization temperature (*T*_cc_), and melting temperature (*T*_m_), as well as the enthalpy change corresponding to the cold crystallization peak (Δ*H*_cc_) and melting peak (Δ*H*_m_) from the second heating ramp. Results showed two different behaviors between feedstocks and 3D printed samples (Figure S2, Supporting Information). In detail, PLA pellet did not reveal any state of crystallization nor melting during the second heating scan. Contrarily, CM showed only a state of melting. This difference could be due to a different thermal history and several processes to obtain the masterbatch, i.e., pelletizing. Furthermore, the absence of the melting peak indicates that the cooling rate applied was too fast for PLA polymer chains to reorganize, preventing recrystallization and melting^31^ (Figure S2a, Supporting Information). Regarding the 3D printed samples, the temperatures recorded in the different states (*T*_g_, *T*_cc_, and *T*_m_) did not report any trend and the difference between the three formulations is almost negligible. The results are consistent with previous examples from literature focused on 3D printing filaments.^30,32^ To summarize, SCGs show limited effects on the thermal behavior of PLA/SCGs composites, indicating that the thermal properties of the matrix and composite formulations are comparable.

### Crystallization Behavior

X-ray diffraction tests investigated the crystallization behavior of 3D printed samples and the influence of SCGs on the polymer matrix. In general, PLA manifests a broad hump of crystallinity in the range of 2θ = 10–25°, typical of amorphous polymers.^32,33^ This fact is confirmed by the results of the 100PLA0CM formulation. Similarly, the 3D printed formulations showed the same range (Figure S3, Supporting Information). The major crystalline peak of the 100PLA0CM, 95PLA5CM, and 90PLA10CM samples was reached at 2θ = 14.9°; 17.3°; 15.6° respectively. Furthermore, the peak intensity of the three formulations ranges around similar values, confirming the limited influence of SCGs on the crystallinity of the 3D printed materials (Table S1, Supporting Information).

### Rheological Behavior

**Figure 1:**
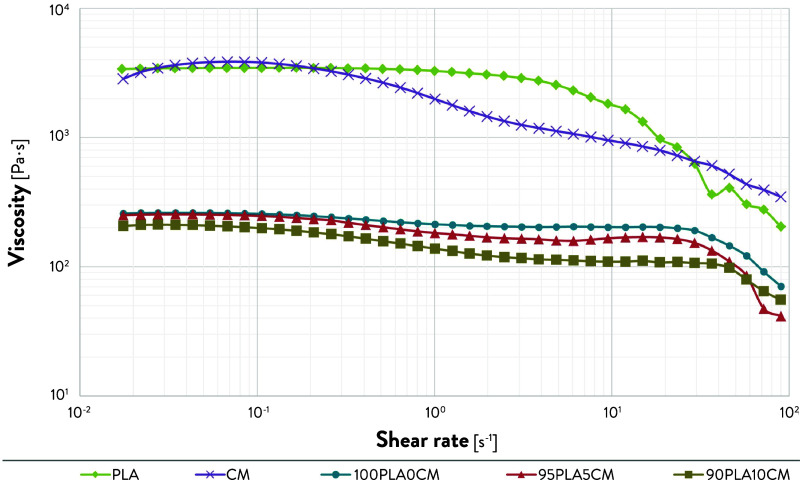
Log–log plot of viscosity as a function of the shear rate of PLA pellets, CM pellets, and the three 3D printed formulations, i.e., 100PLA0CM, 95PLA5CM, and 90PLA10CM.

The rheological behavior of PLA, CM, and the three composite formulations was studied at 190 °C to understand the printability in the extruder chamber. The flow stress ramp curves were obtained by plotting the viscosity as a function of the shear rate on a log–log graph (Figure 1). The curves are non-Newtonian, with starting linear behavior and ending with a shear-thinning region. The viscosity of PLA pellet decreases by 1 order of magnitude at shear rates of 10^2^ s^–1^. Furthermore, CM shows a pseudoplastic behavior, explained by a slight increase in viscosity at lower shear rates with a gradual decrease at shear rates greater than 10^–1^ s^–1^. A significant difference in the viscosity is visible after the 3D printing process. The printed samples already had one cycle of extrusion, and they are therefore no longer considered virgin material; hence, the viscosity decreased about 1 order of magnitude. Although this result shows the influence of a single extrusion process on the viscosity, it also validates using pellet-based feedstock in a single screw extrusion system to avoid further extrusion steps, i.e., to produce 3D printing filaments.

Table 3 shows the zero shear viscosity (η_0_) and the viscosity at a shear rate (γ̇) equal to 27.5 s^–1^, simulating the conditions in the extruder chamber.^34^ A decrease in viscosity is noticeable when the CM weight percentage is increased in the 3D printed formulations. Specifically, the zero shear viscosity decreased by 18%, whereas the viscosity before the shear thinning decreased by 44% from the 100PLA0CM to 90PLA10CM samples. This result indicates a good printability of PLA/SCGs composites since lower viscosity values at higher shear rates decrease the pressure at the nozzle during the extrusion, leading to an easier 3D printing process.^35^ Moreover, a decrease in viscosity improves the consistency of the extrusion flow of the polymer and enhances the coalescence of the extrudate. This homogeneous deposition results in improved layer adhesion and reduced interlayer porosities.^13,36,37^

**Table 3:** Zero Shear Viscosity Values from the Log–Log Plot of Viscosity and Viscosity Values when Shear Rate = 27.5 s^–1^ (Corresponding to a 20 mm/s Feed Rate)

sample		η_0_ (Pa·s)	viscosity when γ̇ = 27.5 s^–1^(Pa·s)
granulated material (pellets)	PLA	3401.9	1820.1
	CM	3787.5	949.5
3D printed material	100PLA0CM	261.9	202.5
	95PLA5CM	255.7	166.1
	90PLA10CM	213.9	109.5

### Mechanical Properties

**Figure 2:**
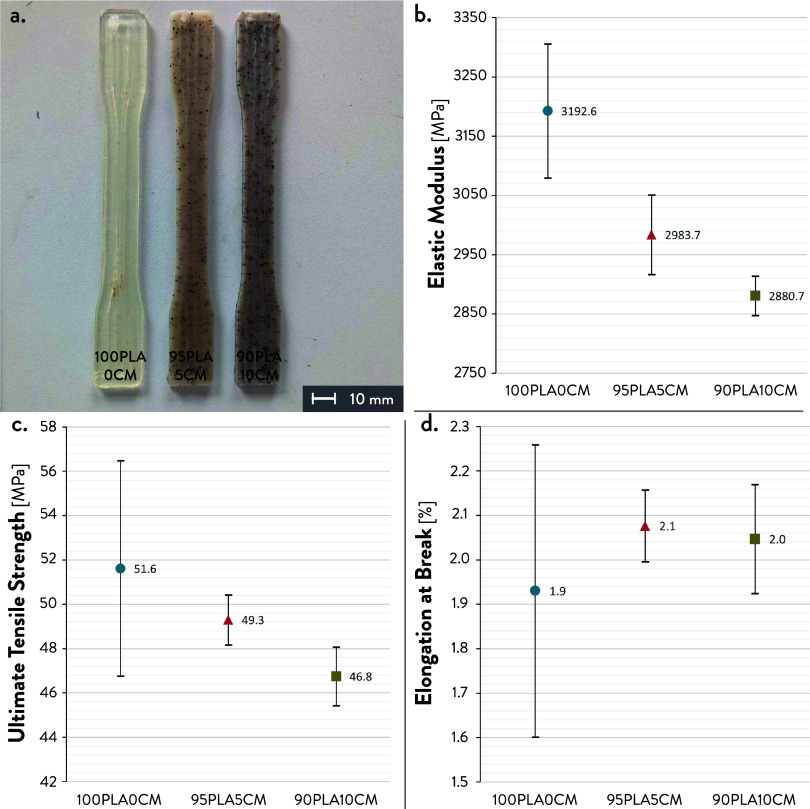
Tensile tests of the three 3D printed material formulations: (a) 3D printed dog bone-shaped specimens;^29^ (b) elastic modulus; (c) tensile strength; and (d) elongation at break.

Tensile tests were performed to evaluate the influence of SCGs on the mechanical properties of the formulations. Table 4 resumes the values of the elastic modulus (*E*), the ultimate tensile strength (σ_m_), and the elongation at break (ε_b_) from the experimental stress–strain curves (Figure S4, Supporting Information). The samples of the three formulations (Figure 2a) showed a brittle failure, as visible from the values of ultimate tensile strength, fracture strength (σ_b_), elongation at maximum strength (ε_m_), and elongation at break (Table S2, Supporting Information). According to Figures 2 and S5 (Supporting Information), 100PLA0CM shows the highest variability, especially for ultimate tensile strength and elongation at break, as shown from the standard deviations. On the contrary, PLA/SCGs formulations show lower variability, indicating greater consistency of the extrudate during the deposition. This result confirms a better printability due to the viscosity reduction from the use of SCGs in the 3D printed formulations. In detail, their presence facilitates the melt flow of the extrudate, hence increasing the diffusion with the interface of the previous layers.^38^ This fact leads to better interlayer adhesion and reduced internal pores and defects, improving the reproducibility of the results compared to the PLA matrix.^13,36^

**Table 4:** Values of Experimental Elastic Modulus, Ultimate Tensile Strength, and Elongation at Break

sample	*E* (MPa)	σ_m_ (MPa)	ε_b_ (%)
100PLA0CM	3192.6 ± 113.2	51.6 ± 4.9	1.9 ± 0.3
95PLA5CM	2983.7 ± 66.9	49.3 ± 1.1	2.1 ± 0.1
90PLA10CM	2880.7 ± 33.3	46.8 ± 1.3	2 ± 0.1

Figure 2b shows a decreasing trend of the elastic modulus when increasing the CM weight percentage, with a reduction of ∼10% compared to neat PLA. The values of ultimate tensile strength (Figure 2c) of the different formulations remain quite constant, with mean values of ∼49 and ∼47 MPa when adding 5 and 10 wt % of CM (95PLA5CM and 90PLA10CM). Similarly, elongation at break (Figure 2d) shows comparable results for the two PLA/CM formulations, i.e., 2.1% (95PLA5CM) and 2% (90PLA10CM). Although SCGs make the 3D printed formulations less stiff compared to PLA, higher values of elastic modulus, ultimate tensile strength, and elongation at break were obtained compared to previous works from the literature.^14,32^ This fact is consistent with the results from the thermal characterization, showing no significant degradation and better printability due to the decrease in viscosity. Using pellet feedstock reduced the processing steps to obtain the raw material, i.e., extruding 3D printing filament, limiting the thermomechanical degradation of a further extrusion cycle on the mechanical properties.^18^

### Optical Microscopy

**Figure 3:**
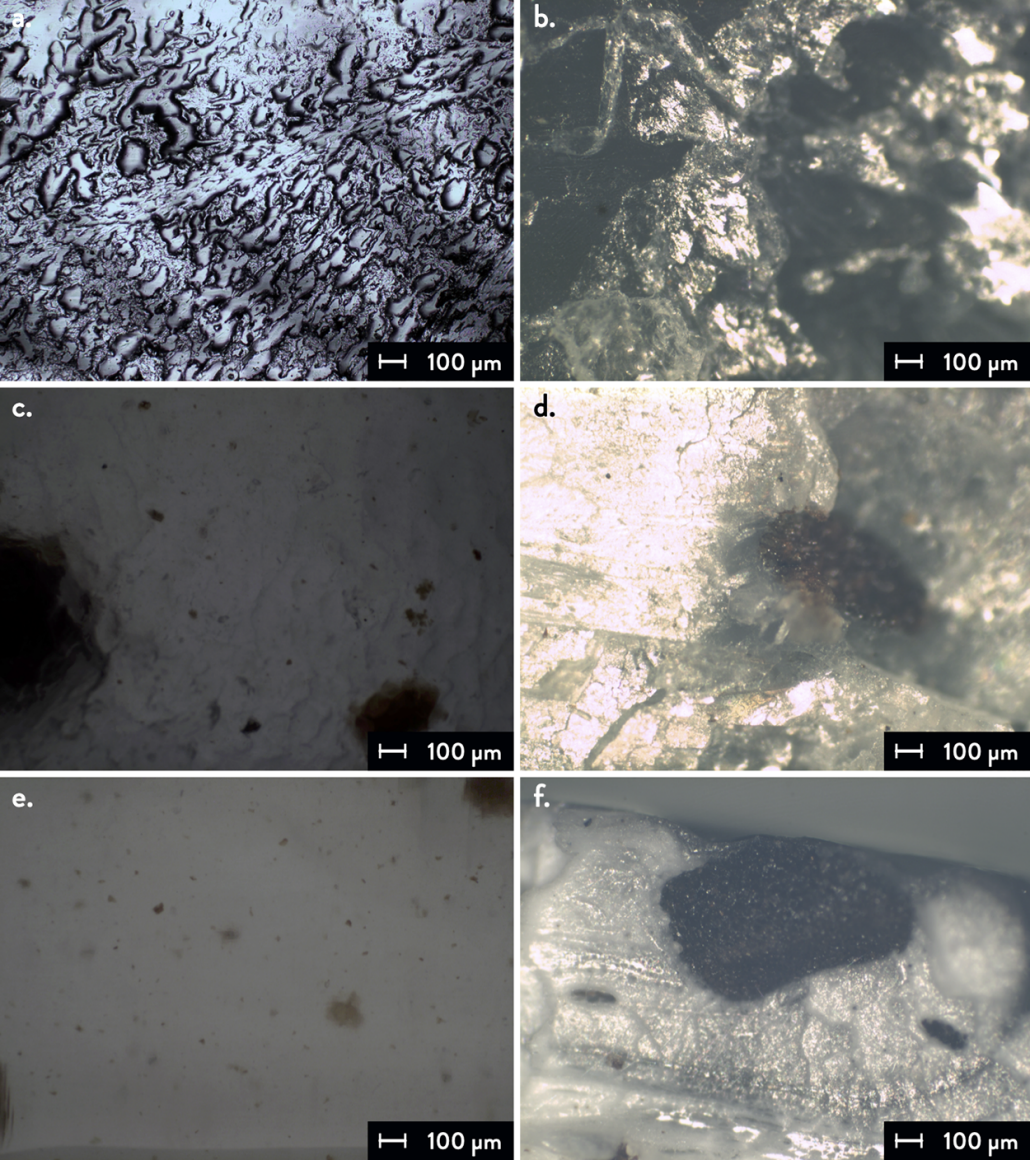
Optical microscopy images of the 3D printed specimens: (a) sample surface and (b) fracture cross section of 100PLA0CM; (c) sample surface and (d) fracture cross section of 95PLA5CM; (e) sample surface and (f) fracture cross section of 90PLA10CM.

Optical microscopy images were taken on the tensile specimens to evaluate the morphology of the 3D printed samples. Figure 3 shows the sample surfaces and the fracture cross-sectional areas of the three formulations, i.e., 100PLA0CM, 95PLA5CM, and 90PLA10CM. An increase in the SGCs content is visible from the micrographs of the sample surfaces (Figure 3a,c,e), together with inhomogeneity in their dispersion into the PLA matrix from the fracture cross section (Figure 3b,d,f). The fracture cross sections show a variable granulometry of SCGs, reaching a maximum dimension of 900 μm (Figure S6, Supporting Information). Since the filler particle size of 3D printable materials with biomass scraps or byproducts usually ranges between 50 and 500 μm, this grain size can cause clogging and affect the composite properties.^22^ However, large-format 3D printers such as the one used in this study may prevent clogging and improve the consistency of the extrusion paths because of large nozzle diameters.

### 3D Printing and Application Case Study

According to the 3D printed dog bone specimens used for the tensile tests (Figure 2a), the processability of the different material formulations is comparable in terms of the overall quality of the final part. First, the samples were fabricated with the same feed rate as in Table 1, indicating a limited influence of SCGs on the extrudability of the PLA/SCGs formulations. Moreover, no clogging or failures occurred by using the formulations with the FGF extruder system, obtaining a consistent extrudate during the 3D printing process, thanks to the selected nozzle diameter. Compared to the 100PLA0CM samples, the specimens made with PLA/SCGs formulations show less visible pores and defects (Figure 2a), supporting the previous results from the characterization.

**Figure 4:**
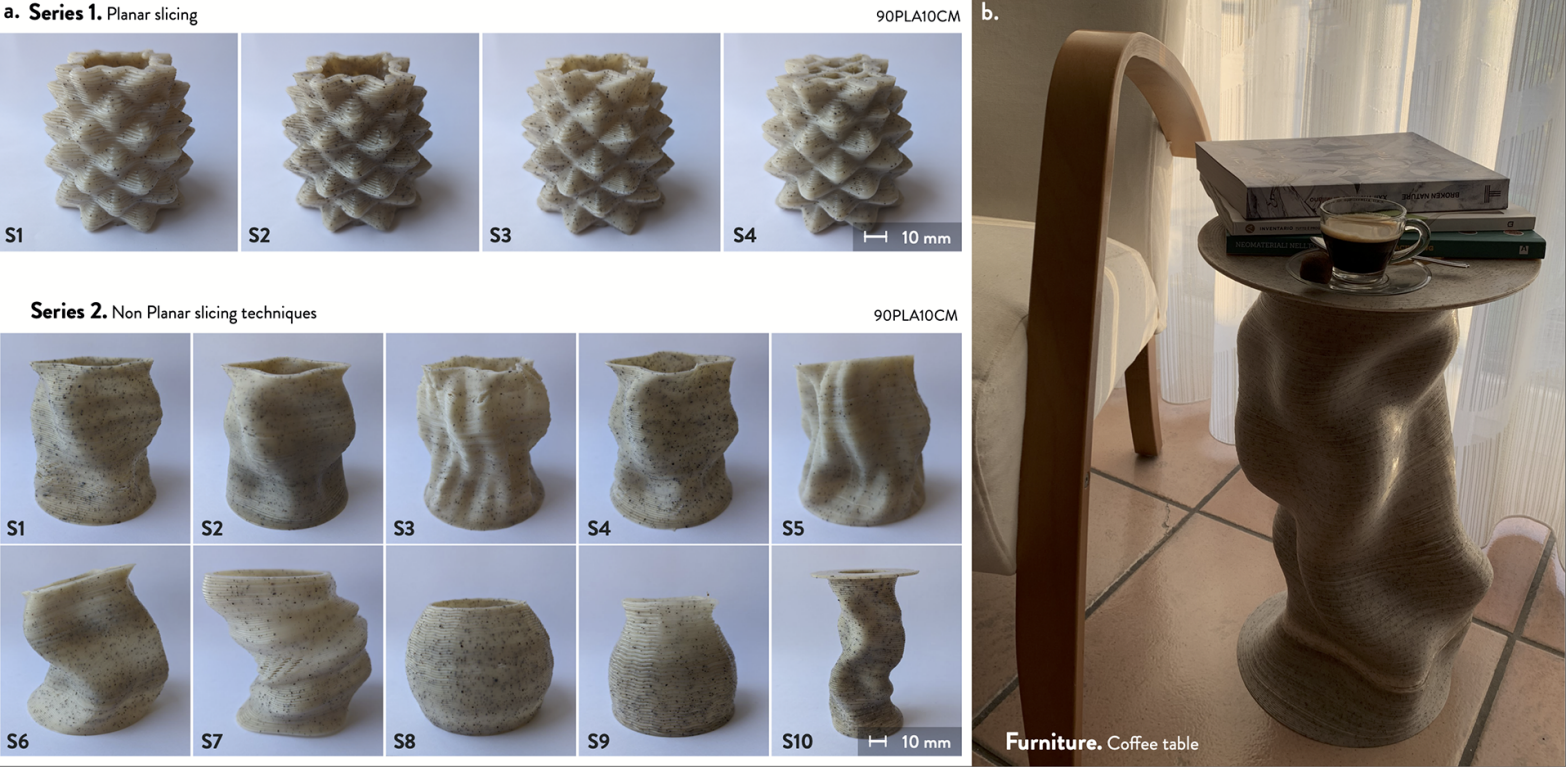
3D printed parts: (a) samples obtained with planar and nonplanar slicing and (b) coffee table (1:1 3D model of furniture product application).

3D printed samples were successfully fabricated using the 10 wt % of CM (90PLA10CM) formulation. This formulation was selected by considering the overall quality of the previous 3D printed characterization specimens in terms of consistency of the extruded paths as well as the maximization of the biomass scraps into the formulation for potential real-world applications. As visible in Figure 4a, the first set of samples was 3D printed with conventional slicing tools, resulting in four samples with different layer heights, i.e., 0.7–1.2 mm, and feed rate, which means from 15 to 30 mm/s, also using 20% infill (Table S3, Supporting Information). The second set of samples was fabricated by using nonplanar slicing techniques, i.e., using nonlinear movements of the extruder head on the *z*-axis,^39^ achieving complex geometries and patterns (Table S4, Figure S7, Supporting Information). The 3D printed samples exhibit homogeneous paths without defects or significant under- or overextruded portions in most cases. Furthermore, no clogging occurred during their fabrication, including at higher feed rates or nonplanar curvature angles in the *z*-axis direction, i.e., 30 mm/s and 32° (Figure 4a,b). These results demonstrate the feasibility of using PLA/SCGs feedstocks with large-format 3D printers.

A potential product in the furniture sector was designed and 3D printed with the 90PLA10CM formulation. The coffee table (Figure 4b) represents a complex structure made with nonplanar slicing and PLA/SCGs through an LFAM FGF 3D printing system. Small batches of customized products can be locally produced to exploit these materials and technologies connected to abundant biomass scraps such as furniture for private and public spaces. Other sectors may be considered to further enlarge the range of potential applications, i.e., lamps, accessories, and interior elements. Applications for high-performance sectors, such as sports or technical equipment, should rely on biomass-based polymer composites with higher strength-to-weight ratios, i.e., natural fiber-reinforced polymers.^22^ In addition, some applications may require further postprocessing to reduce the staircase effect from big nozzle diameters, increasing the fabrication times and costs. Finally, understanding the lifecycle of these products represents a crucial aspect, especially considering possible circular economy strategies when reaching their first end-of-life. Despite the possible reuse of the 3D printed parts, material recyclability can still be challenging due to the heterogeneity of PLA/SCGs composites.^40^ From the literature, mechanical recycling processes can be used to obtain secondary raw materials, also for AM, to extend the use of material resources,^22,41,42^ according to the principles of circular economy.^8^ Studying biodegradability and compostability can also provide future options for dealing with the end-of-life of biobased composites filled with biomass byproducts, such as PLA/SCG formulations.^22^ However, this coffee table contributes to fostering the scaling up of biomass-filled material formulations, validating the use of PLA/SCG composites for real applications.

## Conclusions

This work reported the characterization of a PLA/SCG composite feedstock for applications with an LFAM pellet extrusion system. According to TGA and DSC, PLA/SCGs formulations are thermally stable during the 3D printing process with minimal degradation of SCGs at typical 3D printing temperatures of PLA, i.e., ∼190 °C. The influence of SCGs on the crystallinity of 3D printed materials is limited, with similar ranges of peak intensity from XRD. The formulations exhibit a non-Newtonian behavior, and their viscosity decreases by increasing the SGCs weight percentage, improving printability and layer adhesion. Tensile tests are consistent with these results, showing higher values of ultimate tensile strength and elongation at break compared with the state-of-the-art. Although some variability in SCGs dispersion and granulometry was detected through micrographs, using pellet feedstock limited the thermomechanical degradation due to further raw material processing, i.e., extruding filaments. Different sample batches with complex geometries or nonplanar slicing techniques were successfully 3D printed, including a large-scale product in the furniture sector. Further work should be done to investigate the biodegradability and recyclability of the formulations and other application fields as well as deepen the characterization of PLA/SCG composites. However, LFAM and FGF systems represent a potential scaling-up solution for polymer-based composites from biomass byproducts, such as PLA/SCG formulations.

## Supporting Information Available

Additional information on the characterization of PLA pellets, CM pellets, and the three PLA/CM formulations (DSC, TGA, XRD, and tensile tests) and the 3D printed samples (PDF)

## Abbreviations

AM: Additive Manufacturing
CM: Coffee Masterbatch
DSC: Differential Scanning Calorimetry
FDM: Fused Deposition Modeling
FFF: Fused Filament Fabrication
FGF: Fused Granular Fabrication
LFAM: Large-Format Additive Manufacturing
PLA: Polylactic Acid
PE: Polyethylene
PP: Polypropylene
SDGs: Sustainable Development Goals
TGA: Thermogravimetric Analysis
XRD: X-ray Diffraction.
