# Supplementary material for: PLA Feedstock Filled with Spent Coffee Grounds for New Product Applications with Large-Format Material Extrusion Additive Manufacturing
Source: ACS Omega. 2024 Feb 1;9(6):6423–31. doi: 10.1021/acsomega.3c05669 (PMC10870276; doi:10.1021/acsomega.3c05669)
Supplement: Supplementary file 1 — ao3c05669_si_001.pdf [file ao3c05669_si_001.pdf]

PLA feedstock filled with spent coffee grounds for new product applications with large-format Material Extrusion Additive Manufacturing

Supporting Information

Martina Paramatti †,‖, Alessia Romani †,‡,‖, Gianluca Pugliese †‡, Marinella Levi †*

‖ M.P. and A.R. are equally contributed to the work.

† Department of Chemistry, Materials, and Chemical Engineering "Giulio Natta", Piazza Leonardo Da Vinci 32, 20133 Milano, Italy

‡ Design Department, Via Durando, 20158 Milano, Italy

†‡ LowPoly SL, Avenida Real de Pinto 91, Nave 07, 28021 Madrid, Spain

* Corresponding author: alessia.romani@polimi.it (Alessia Romani).

Note S1. Additional information from material characterization (TGA, DSC, XRD, Tensile tests)

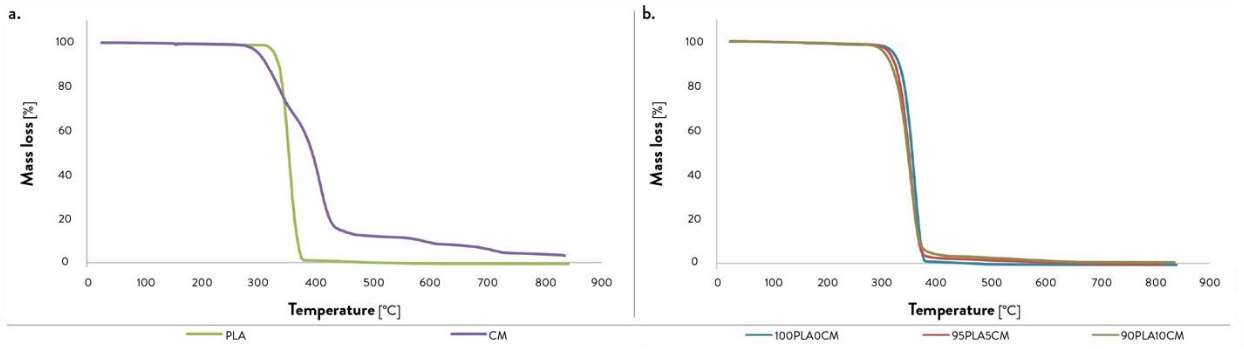

Figure S1. TGA curves of: (a) PLA pellet and CM; and (b) the three 3D printed formulations, i.e., 100PLA0CM, 95PLA5CM, and 90PLA10CM.

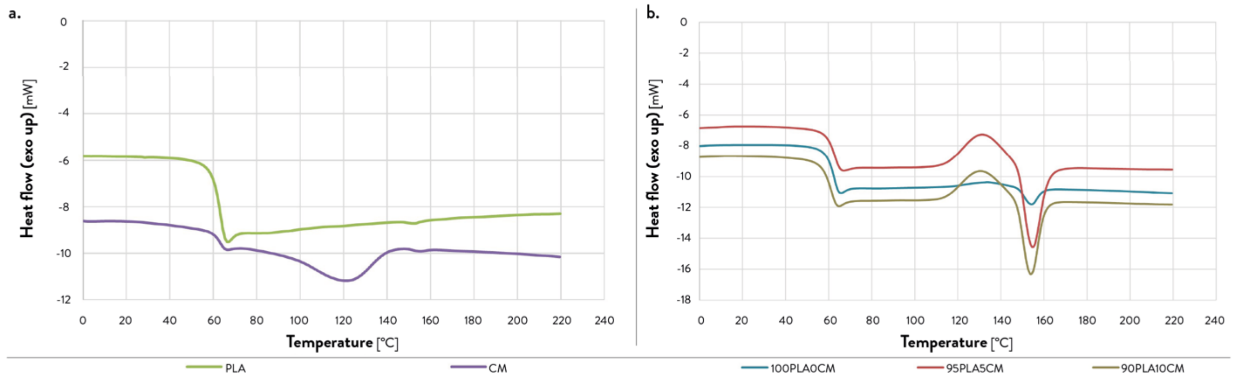

Figure S2. DSC curves of: (a) PLA pellet and CM; and (b) the three 3D printed formulations, i.e., 100PLA0CM, 95PLA5CM, and 90PLA10CM.

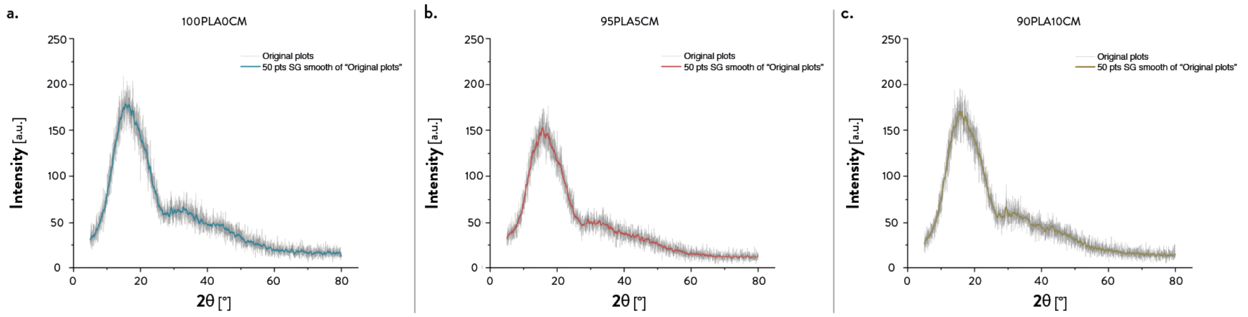

Figure S3. XRD patterns of the three 3D printed formulations: (a) 100PLA0CM; (b) 95PLA5CM; and (c) 90PLA10CM.

Table S1. Values of intensity and the major crystalline peak of the three 3D printed formulations, i.e., 100PLA0CM, 95PLA5CM, and 90PLA10CM.

Sample	*Intensity (a.u.)*	*2θ (°)*
100PLA0CM	209	14.9
95PLA5CM	177	17.3
90PLA10CM	196	15.6

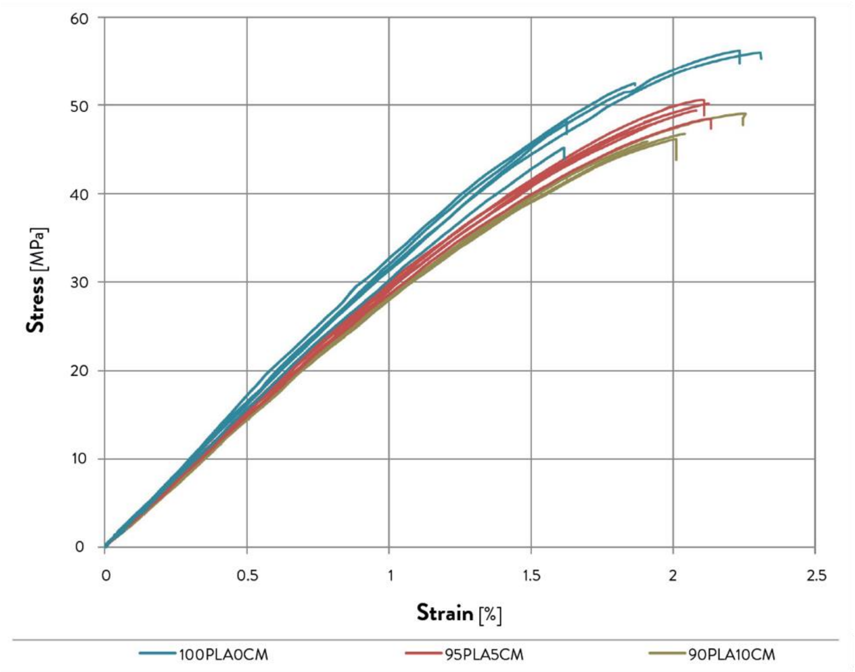

Figure S4. Stress-strain curves from the three batches of the 3D printed tensile specimens: 100PLA0CM (blue), 95PLA5CM (red), and 90PLA10CM (green).

Table S2. Values of experimental elastic modulus, ultimate tensile strength, fracture strength, elongation at maximum strength, and elongation at break.

Sample	*E* (*MPa*)	σ_m (*MPa*)	σ_b (*MPa*)	ε_m (*%*)	ε_b (*%*)
100PLA0CM	3192.6 ± 113.2	51.6 ± 4.9	50.6 ± 5.1	1.9 ± 0.3	1.9 ± 0.3
95PLA5CM	2983.7 ± 66.9	49.3 ± 1.1	48.7 ± 1.1	2.1 ± 0.1	2.1 ± 0.1
90PLA10CM	2880.7 ± 33.3	46.8 ± 1.3	46.0 ± 1.5	2 ± 0.1	2 ± 0.1

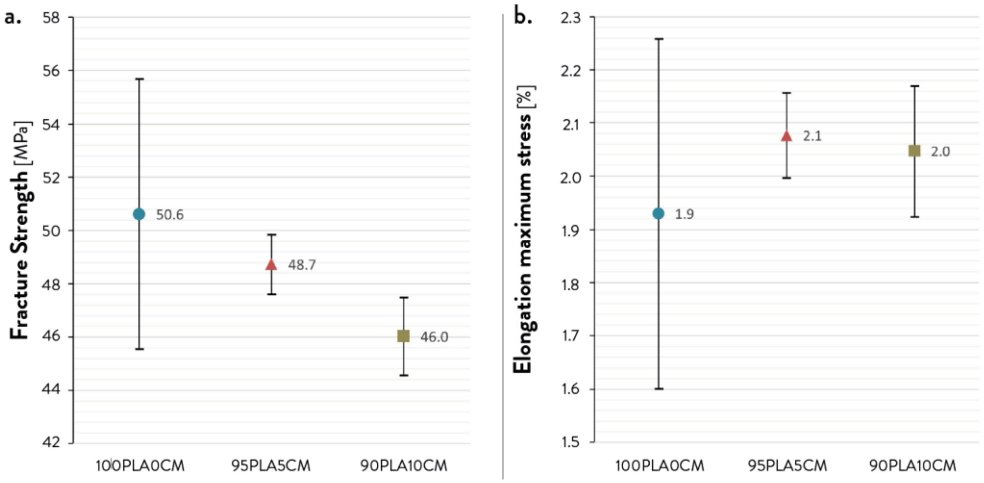

Figure S5. Comparison of the experimental values from the tensile tests: (a) fracture strength and (b) elongation at maximum stress.

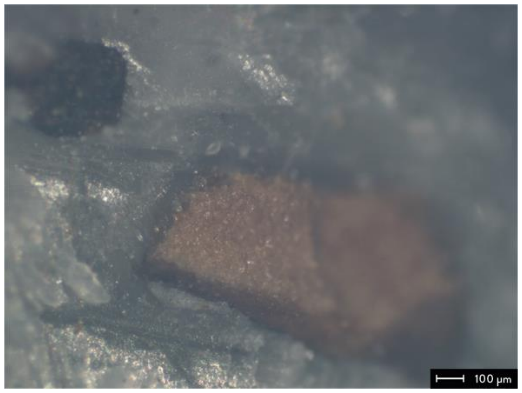

Figure S6. Optical microscopy image of a SCG grain on the 3D printed specimen surface.

Note S2. 3D printed samples with planar and nonplanar slicing techniques.

The 3D printed samples were used to demonstrate the feasibility of complex geometries using the formulation with 10% of Coffee Masterbatch (CM), i.e., 90PLA10CM, with the LFAM (Large-Format Additive Manufacturing) FGF (Fused Granular Fabrication) system.

The first set of samples was 3D printed with conventional slicing process, i.e., planar slicing. The geometry was designed to assess the shape retention of surface textures with LFAM systems. A revolution-like shape with an embossed and engraved diamond texture was selected for the qualitative comparison of the printability.

At least two samples of four different combination of parameters were 3D printed for this sample batch. Table S3 resumes the main parameters of the four samples, namely S1, S2, S3, and S4. In short, layer height was set from one-fifth to one-fourth of the nozzle diameter (3 mm). Feed rate was incremented from 15 to 30 mm/s according to the results from the flow ramp stress test, which indicate a decrease in viscosity and a better printability at shear rates equal to 20 mm/s. Finally, one perimeter was set during the gcode creation for samples from S1 to S3 to assess the shape retention of the overhangs from the texture surface. The 20% infill was added to S4 to check its potential use in complex geometries.

The second set of samples was fabricated with nonplanar slicing techniques. Contrarily to common slicing tools, nonplanar slicing uses curved extrusion paths in the z-axis, corresponding to nonlinear movements of the extruder head in the same direction[1,2]. Five different geometries were designed to evaluate the use of nonplanar slicing with LFAM FGF systems and PLA/Spent Coffee Ground (SCG) composites, resulting in ten samples combining three different variables, as shown in the scheme of Fig. S7. The first variable, layer height, ranged from about one-sixth to one-fourth of the nozzle diameter (3 mm), trying to reach higher shape fidelity in the z-axis. The second variable, slicing technique, focuses on the approach used for nonplanar slicing. In detail, "Nonlinear paths" correspond to a variation in the z-axis of the extrusion path, which means the 3D printed perimeter; "Nonplanar slicing" creates a bending effect on the different layers, modifying the bending angle with respect to the building plate (xy plane); "Nonlinear patterns" modify the layer-by-layer appearance in z-axis by adding a pattern with nonlinear paths; "Nonplanar combination" merges the first two approaches in a single model, combining nonlinear paths to bending effect. The third variable, curvature angle, refers to the maximum angle variation achieved with nonplanar slicing techniques, affected by the geometry and components of the extruder head of Delta Wasp 3MT Industrial. A maximum of 32° was achieved in this work.

Table S4 resumes the main parameters of the ten samples, from S1 to S10. Considering the complexity of the geometry, feed rate was kept constant to 10 mm/s. One perimeter was set for each sample to facilitate the comparison between different nonplanar slicing approaches (variable 2) and curvature angles (variable 3). The geometries were 3D modelled in Grasshopper as parametric shapes defined by isocurves, which means by directly modelling the gcode path [2,3].

Sample S10 of the second set corresponds to the 1:10 scaled version of the product application (coffee table) since it represents the synthesis of the nonlinear and nonplanar approaches.

Table S3. Slicer parameters of the 3D printed samples (Series 1).

Samples	Formulation	Layer Height	Feed rate	3D printing mode
	(mm)	(mm)	(mm/s)	(//)
S1	90PLA10CM	1.2	15	One shell/perimeter
S2	90PLA10CM	1.0	20	One shell/perimeter
S3	90PLA10CM	0.7	30	One shell/perimeter
S4	90PLA10CM	0.7	15	One shell/perimeter<br>20% infill

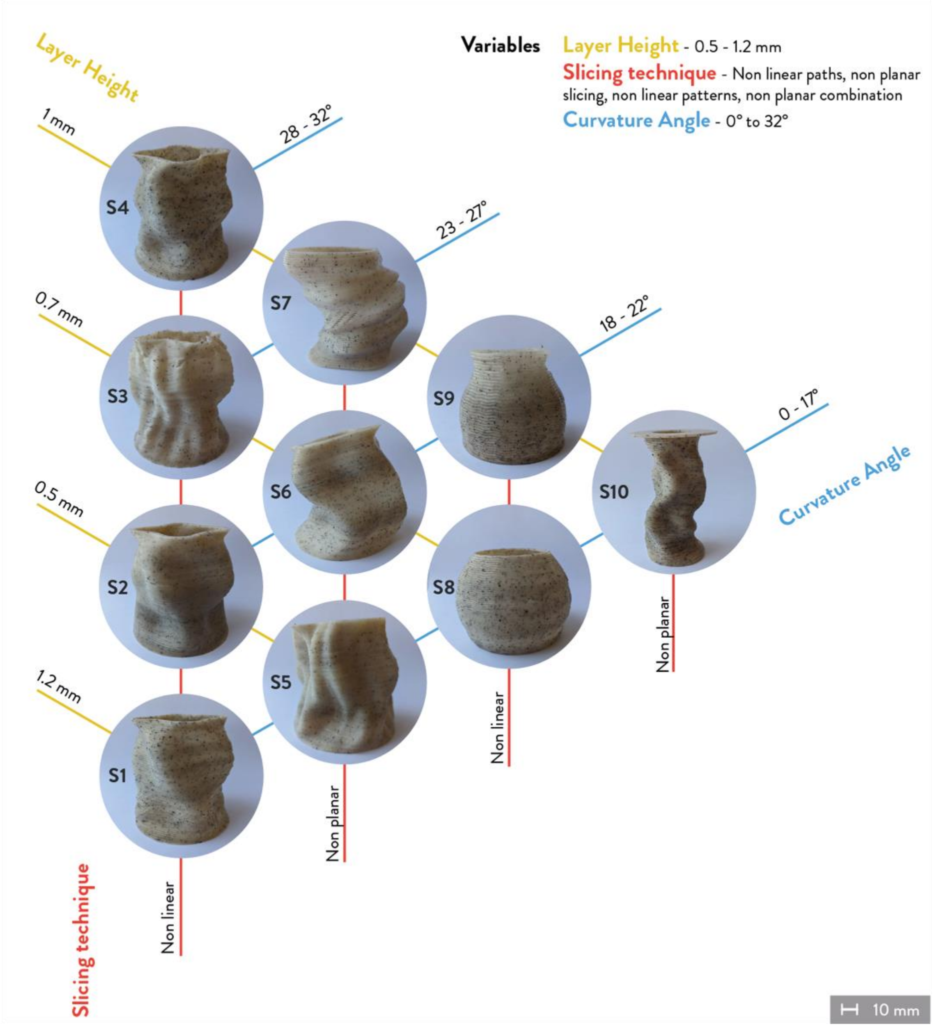

Figure S7. Variation of the main parameters of the 3D printed samples (Series 2).

Table S4. Slicer parameters of the 3D printed samples (Series 2).

Samples	Formulation (mm)	Layer Height (mm)	Slicing technique (//)	Curvature angle (slicing) (°)
S1	90PLA10CM	1.2	Non linear paths	0-17
S2	90PLA10CM	0.5	Non linear paths	18-22
S3	90PLA10CM	0.7	Non linear paths	23-27
S4	90PLA10CM	1	Non linear paths	28-32
S5	90PLA10CM	0.5	Non planar slicing	0-17
S6	90PLA10CM	0.7	Non planar slicing	18-22
S7	90PLA10CM	1	Non planar slicing	23-27
S8	90PLA10CM	0.7	Non linear surface patterns	0-17
S9	90PLA10CM	1	Non linear surface patterns	18-22
S10	90PLA10CM	1	Non planar slicing and non linear paths	0-17

Additional References.

(1) Nisja, G. A.; Cao, A.; Gao, C. Short Review of Nonplanar Fused Deposition Modeling Printing. *Mat. Des. Process. Commun.* **2021**, mdp2.221. https://doi.org/10.1002/mdp2.221.
(2) Nayyeri, P.; Zareinia, K.; Bougherara, H. Planar and Nonplanar Slicing Algorithms for Fused Deposition Modeling Technology: A Critical Review. *Int J Adv Manuf Technol* **2022**, *119* (5), 2785–2810. https://doi.org/10.1007/s00170-021-08347-x.
(3) Cuevas, D. D. G.; Pugliese, D. G. *Advanced 3D Printing with Grasshopper®: Clay and FDM*; Independently published: Wrocław, 2020.
